# The Statistics of Urban Scaling and Their Connection to Zipf’s Law

**DOI:** 10.1371/journal.pone.0040393

**Published:** 2012-07-18

**Authors:** Andres Gomez-Lievano, HyeJin Youn, Luís M. A. Bettencourt

**Affiliations:** 1 School of Human Evolution and Social Change, Arizona State University, Tempe, Arizona, United States of America; 2 Santa Fe Institute, Santa Fe, New Mexico, United States of America; University of Namur, Belgium

## Abstract

Urban scaling relations characterizing how diverse properties of cities vary on average with their population size have recently been shown to be a general quantitative property of many urban systems around the world. However, in previous studies the statistics of urban indicators were not analyzed in detail, raising important questions about the full characterization of urban properties and how scaling relations may emerge in these larger contexts. Here, we build a self-consistent statistical framework that characterizes the joint probability distributions of urban indicators and city population sizes across an urban system. To develop this framework empirically we use one of the most granular and stochastic urban indicators available, specifically measuring homicides in cities of Brazil, Colombia and Mexico, three nations with high and fast changing rates of violent crime. We use these data to derive the conditional probability of the number of homicides per year given the population size of a city. To do this we use Bayes’ rule together with the estimated conditional probability of city size given their number of homicides and the distribution of total homicides. We then show that scaling laws emerge as expectation values of these conditional statistics. Knowledge of these distributions implies, in turn, a relationship between scaling and population size distribution exponents that can be used to predict Zipf’s exponent from urban indicator statistics. Our results also suggest how a general statistical theory of urban indicators may be constructed from the stochastic dynamics of social interaction processes in cities.

## Introduction

The search for a general multidisciplinary science of cities is a fundamental scientific problem with strong roots in economics [1], [2], sociology [3]–[5], urban planning and architecture [6], [7]. As human populations become increasingly urban the quantification of general insights and solutions that transcend each particular place is increasingly necessary and would have important consequences for our fundamental understanding of human societies and for urban planning and policy [8].

Cities should be regarded primarily as dynamical social networks, constantly changing in terms of their composition and interactions. Consequently, urban indicators, denoted by 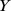, and population 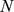, should be treated in general as stochastic variables. More specifically, there are practical circumstances when a full statistical approach to urban quantities becomes necessary. For example, a statistical treatment of urban indicators is essential when average characterizations is insufficient because of the granularity that arises when dealing with small integer numbers in 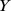 (or in 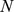). In such extreme regimes we may investigate if and to what extent urban scaling laws apply and how they may emerge in the limit of large numbers, when 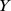 can be thought of as an effectively continuous variable.

In order to probe urban indicators that show granularity and a large level of temporal and geographic variation we analyze here data on annual homicides in cities of three Latin American countries over a several year period during which national homicide rates have varied substantially. We analyze data from three of the largest nations in Latin America, presently showing some of the highest homicide rates in the world: Brazil, Colombia and Mexico, for which data are available at the municipal level. The number of homicides is a quantity that is widely available at the local level in developed and developing nations. It is thought generally to be reliably reported, notwithstanding some important caveats [9]. For these reasons, we use the annual number of homicides in Latin American cities to develop a statistical approach to urban scaling.

Homicides, as the ultimate expression of violence in human societies, are a widely investigated quantity [10], [11]. Many reasons have been advocated for the rise and fall of homicides in cities throughout the world, especially in the US and Europe [12]. Here it is not our intention to distinguish between these ideas or propose new ones, but to determine general characteristics of the statistics of homicides in connection with the population size of a city. More specifically, our main objective is to establish general properties of the statistics of urban indicators in the limit of high granularity and to investigate if and how urban scaling laws emerge and are related to Zipf’s law for the population size of cities. We expect that such insights should extend to other urban indicators and shed some light on a full statistical theory of cities in terms of their quantitative observable properties.

In the next section we discuss some of the characteristics of the data and our main formal objective, the estimation of the conditional probability density 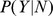 for a particular realization 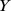 in a city with population 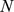. Because no two cities have the same population direct estimation of this probability is impractical so we exploit Bayes’ rule to compute instead  and 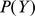. We describe the statistical properties of these two distributions and then derive a closed form for 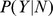. We then show that scaling laws emerge as the expectation value of 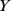 given 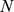 and how knowledge of the conditional distributions and of 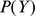 lead to Zipf’s law for the size distribution of cities. Finally, we discuss several qualifications and generalizations of these results and some of their general implications.

## Results

### Scaling Relations and Units of Analysis

We have recently shown [13], [14] that many urban properties 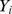 vary, on average, with city population size 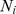 according to a scaling relation.
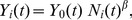
(1)where the subscript 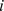 refers to a particular city within an urban system at time 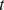, 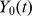 sets the baseline value of 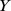 for the urban system and the exponent 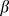 measures the average relative change in 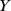 with 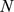, 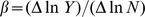. In particular, for socioeconomic quantities such as urban GDP, wages or violent crime 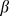 is typically superlinear (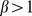), expressing an average per capita increase in these quantities with city size 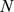. Here we go beyond mean expectations to show how Eq.(1) emerges statistically.

We have also observed that for US metropolitan areas many urban quantities vary only slowly, with most change being due to the temporal variation of 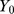 and to the dynamics of population change. This has the consequence that deviations from average scaling - for example in economic quantities or measures of innovation - tend to persist, and sometimes be reinforced, for several decades [14]. Under these circumstances it becomes difficult to observe systematic variations in the statistics of urban metrics, precluding us from eventually establishing the properties of their underlying processes.

To address these points, we introduce here new extensive data sets for homicides in three fast evolving (and developing) nations: Brazil, Colombia and Mexico. These nations are presently among the most violent in the world with registered homicide rates greater than 15 per 100,000 inhabitants, see [10], [11]. Their homicide rates have, in addition, experienced substantial changes over time, both at the national level and in some particular cities. In all three cases the rise in violence, especially in particular cities, has become a major impediment to national economic development and a challenge to international security. Changes in crime rates in these nations, as elsewhere, have been attributed to new initiatives to fight organized crime [15] or to the rise of several organized crime groups and to ‘wars’ between them [16]. Although these and other explanations for the variation of crime in cities have been advanced and are widely discussed in the literature, the evaluation of their relative merits requires, in our opinion, improved statistical models, that quantify and specify the nature of fluctuations and go beyond average rates.

In Brazil, Colombia and Mexico the smallest spatial unit for which data are available is the municipality (*municipio*). Municipios are defined as the smallest administrative units with a local government. Because municipalities partition the entire national territory, their interpretation as urban units is flawed, just as it would be to assume that each county in the US, for example, is a city. Most municipalities consist, in fact, of several human settlements over extensive rural areas. This introduces a limitation in the resolution at the smallest population scales. At the larger population scales we can address this issue because large functional cities (metropolitan areas) are made up of a set of municipalities. Thus, bearing in mind these caveats, we adopt a definition of urban units in terms of metropolitan areas for which an official definition exists, plus the remaining non-metropolitan municipalities. Data sources, definitions and more details are provided in the Methods section.

**Figure 1:**
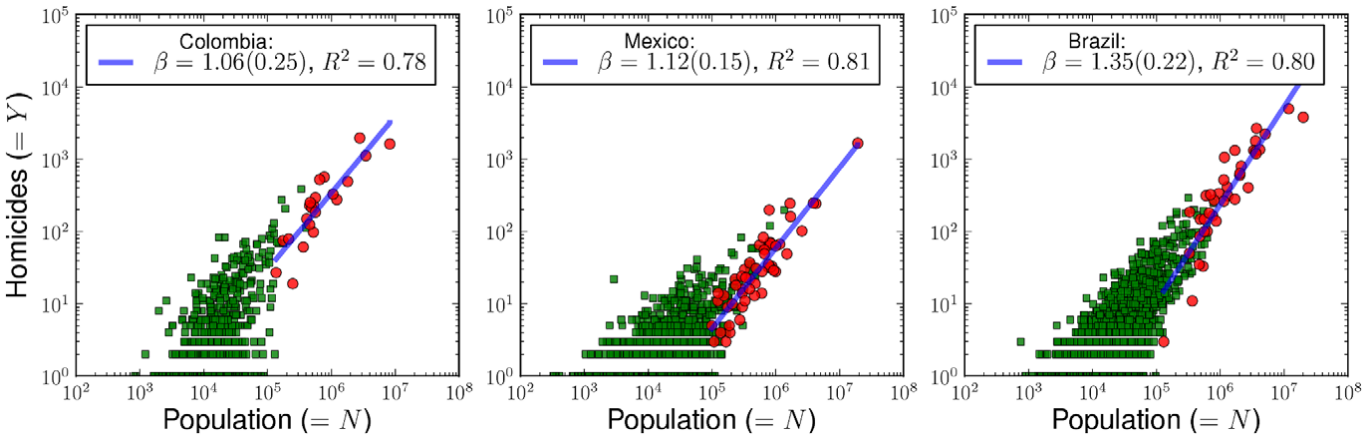
Annual number of homicides in cities of Colombia, Mexico and Brazil versus population size (2007). Large cities are defined in terms of metropolitan areas which are aggregations of municipalities (red circles) while non-metropolitan municipalities are shown separately (green squares). The solid blue line fits only the scaling of homicides for metropolitan areas. Large variations, especially among the smaller population units, and the fact that many municipalities have 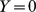 (not shown) prevent a direct scaling analysis. However, it is possible to analyze the data consistently through the estimation of conditional probabilities.

We motivate the need for our statistical study by displaying in Figure 1 the scaling of total homicides versus population size over a single year. The solid line fits the scaling of homicides for metropolitan areas only. Large differences are displayed between municipalities, and our goal is to characterize these fluctuations in a complete framework. We will not discuss the specificities of urban homicides but their general statistical nature, and their relation to scaling and Zipf’s law.

### Bayesian Approach to the Statistics of Urban Indicators

Equation (1) is an average statement that cannot be obeyed exactly in every instance. This is not only because all cities have specific local characteristics and urban indicators fluctuate over time but, more fundamentally, because a continuous scaling relation must break down in the limit of small discrete numbers. The correct statement must then be formulated in probabilistic terms. To do this we think of both 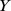 and 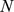 as stochastic variables, and of their values at each particular city and time as statistical realizations. We can then estimate their probability distributions.

This problem is specified in terms of the conditional probability distribution function of 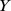, given a city of population 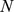, 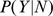. We use Bayes’ rule
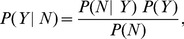
(2)to compute it, given knowledge of the probability distribution 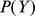 of homicides in cities regardless of their population, and the conditional probability distribution 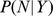 for the population size of cities with a given number of homicides. The denominator is a constant in 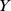 and can be expressed as the trace of the numerator over all values of 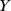. We will return to this point below as 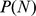 is Zipf’s probability density function for city population sizes.

The reason to estimate 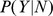 indirectly is motivated primarily by practical considerations. To estimate 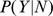 directly we would have to aggregate cities of similar size together into arbitrary discrete size intervals (binning), potentially introducing errors and leading to several additional complications. To avoid this, we exploit to our advantage the granularity of the data as there are substantial numbers of cities with 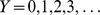 leading naturally to estimates of 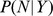.

### Estimating the Distribution of Total Urban Homicides

The distribution of total homicides in cities 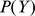 must reflect the fact that urban properties change (super)extensively with population and that there are cities with widely varying sizes. As such we should expect 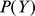 to be a broad distribution. Because of these general facts, power-law probability densities (Zipf or Pareto distributions), are common among urban metrics. More specifically, these distributions account for the fact that a small number of cities are responsible for most homicides and that a large number of cities display only a few. In Mexico, for example, approximately 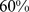 of homicides come from 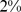 of cities! Similar numbers characterize Colombia and Brazil for the years studied.

In practice, we adopt a common procedure of plotting the complementary cumulative distribution function rather than the probability density function, which avoids the noisy character of the tail for large cities.

**Figure 2:**
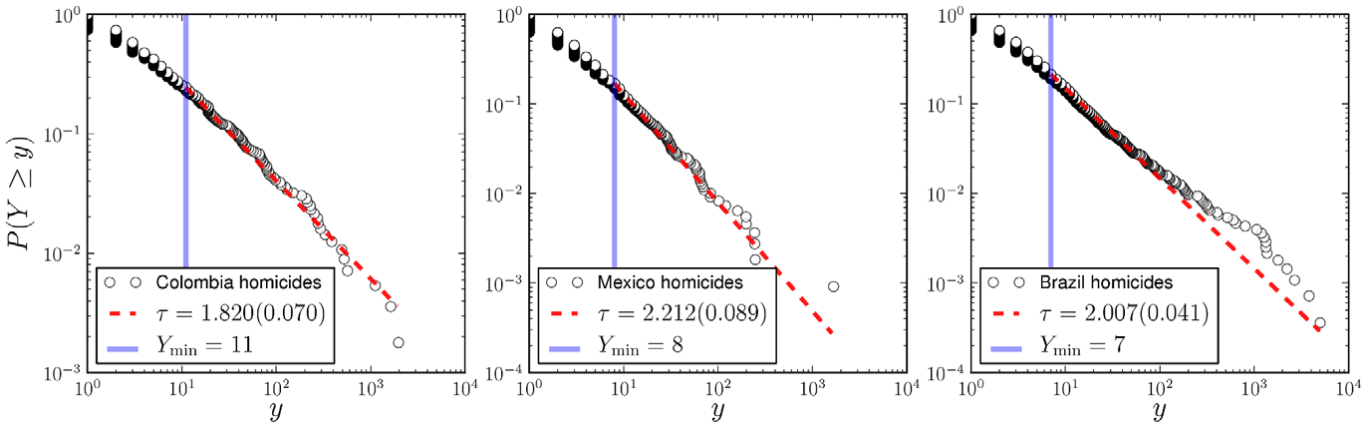
Cumulative normalized distributions of homicides in Colombia, Mexico and Brazil (2007) are well described by power-law distributions. Here we plot not the density function but the complementary cumulative distribution to attenuate the tail fluctuations and ease visual interpretation. Best fits (dashed red line) of the form 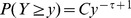 were estimated using the procedure in [17] to the density function (see Methods section). Standard errors are reported in parenthesis. The solid blue line shows the minimum value of 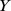 for which a power-law fit holds. While the distribution of total homicides is scale invariant, this is the result of tracing more predictable conditional distributions for each city over a broad distribution of city sizes (see text).

The empirical cumulative distributions of homicides for the year 2007 in Colombia, Mexico and Brazil are shown in Fig. 2. These distributions appear very similar, showing a heavy tail for several decades and an effective lower cutoff for small values of 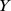. We were unable to reject power-law fits using the procedure of [17]. We assumed the functional form of 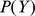 to be
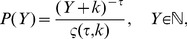
(3)where 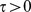 is the power-law exponent and 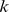 is a positive real number, which allows 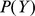 to remain analytic as 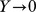. Here
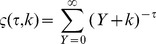
(4)is the generalized or Hurwitz zeta function [18], which ensures the normalization of 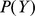 as a discrete variable.

### Estimating 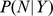 and Deriving 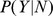


**Figure 3:**
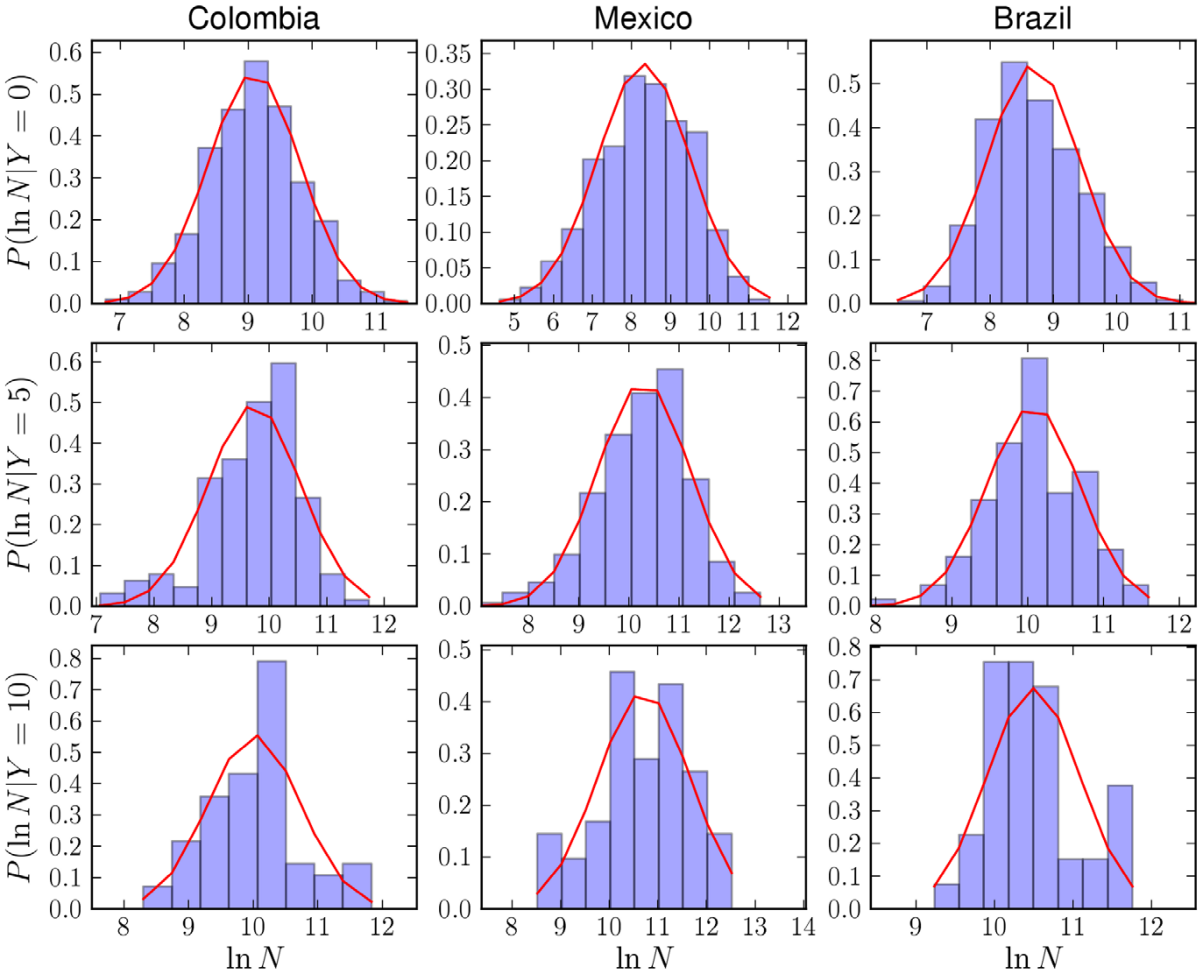
Normalized frequency histograms of the logarithm of city population for varying number of observed homicides *Y*. Each column corresponds to a different country and each row, from top to bottom, corresponds to the values 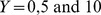 homicides per year. A log-normal distribution (notice the x-axis is expressed in terms of 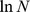) is shown as a solid red line, with parameters obtained via maximum likelihood estimation.

To calculate 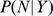 we fix the value of 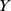 and estimate the probability distribution over population. Fig. 3 shows the histograms of frequencies of homicides for Colombia, Mexico and Brazil, for a range of 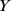. Note that 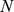 in the x-axis is plotted on a logarithmic scale (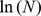). These figures give us an impression of what type of probability distribution describes the data. We observe that all distributions, at each value of 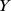, show a distinct peak with definite mean and variance. The null hypothesis of a Poisson distribution was rejected with high confidence by a maximum likelihood method. Instead, these are well fit in terms of a log-normal distribution:
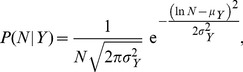
(5)where the subscript in 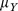 and 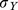 indicates that these parameters are in general functions of 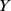.

The shape of this distribution, which we had more implicitly noted in [14] for other quantities, is perhaps curious, first, because it does not conform to the more classic distributions, such as the Gaussian or Poisson, despite the fact we are dealing with count data that are traditionally related to neutral processes like the law of rare events (see [19]). And second, because it states that urban metrics are much more predictable given other variables (here simply population size) than a Zipfian distribution might have lead us to believe. Thus, effectively a Zipf distribution blurs fairly predictable quantities, given 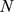, over a broad range of population sizes. Seen from the opposite perspective, log-normal distributions are what we observe if we look at the variables described by a Zipf distribution through a “lens” that allows us to distinguish its many (and widely varying in size) component units (cities).

One drawback of the log-normal distribution is that both 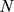 and 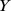 are in reality discrete numbers, whereas the log-normal describes typically a continuous stochastic variable. (Discrete log-normal distributions are sometimes used in the statistics literature, see [20] and references therein). In spite of this property, it is still reasonable to assume that the variation in population is approximately continuous as the minimal values of 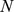 are typically on the order of thousands.

The mean and variance are given by:
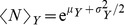
(6)

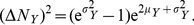
(7)


The maximum likelihood estimators of the log-normal parameters are:
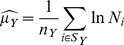
(8)

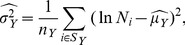
(9)where 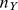 is the number of cities in the set 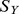 with 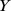 homicides.

**Figure 4:**
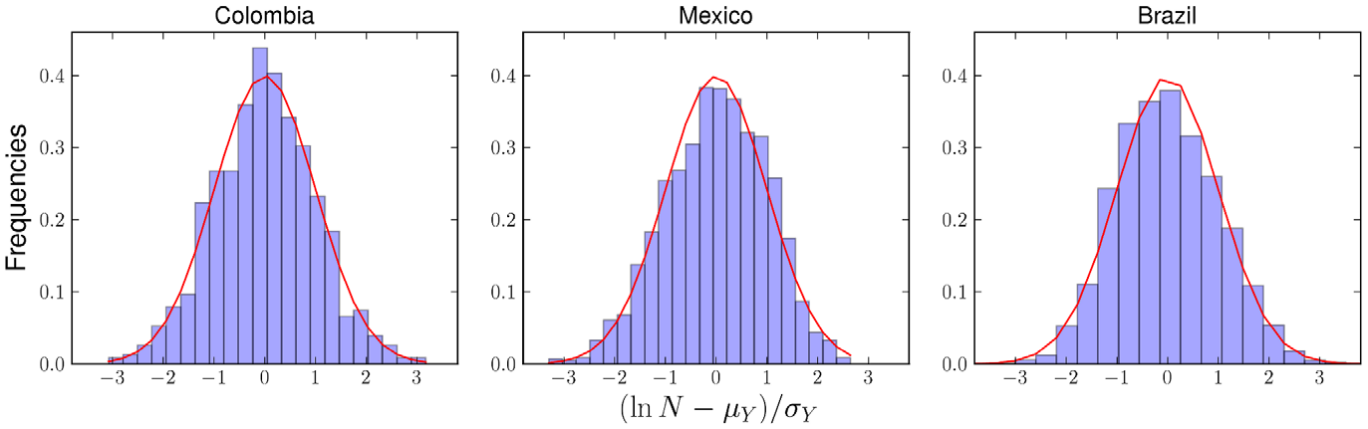
Collapsed histograms of *P*(*N *|* Y*) across values of *Y* in 2007. Log-normal probability density functions for the three nations are shown as solid red lines. This shows that power-law distributions describing total homicides in the urban systems have in fact more predictable statistics when conditioned on city population size.

If the normal distribution holds in terms the logarithmic variables of population given different values of 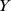, we can collapse the different histograms of Fig. 3 by standardizing log-variables. We achieve this by calculating the maximum likelihood estimators of the mean and variance for every value of 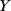, and then plotting in the same histogram the distribution for several values of 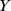. Fig. 4 shows these standardized distributions. This procedure has its limitations due to the fact that as we increase 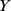, the number of cities decreases, until there is only one city with given 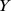 and 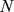 and statistical estimation becomes impossible. Conversely, it has the advantage that the shape of the distribution 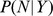 for several values of 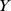 can be displayed in one single figure.

**Figure 5:**
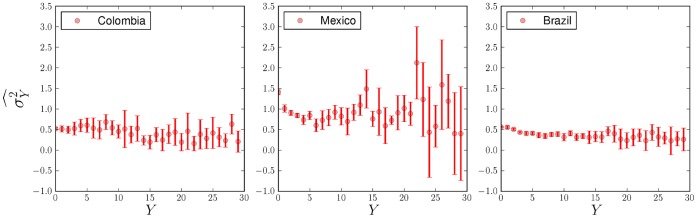
Estimates of 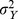 (via maximum likelihood) for different values of 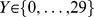, for Colombia, Mexico, and Brazil. A different curve was constructed for every year of the analysis (see Methods). The plots show the average over several years. Error bars represent one standard deviation intervals (67% confidence level). The plots show no clear systematic 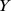-dependence of 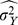. This suggests, in turn, that each country has a characteristic variance of its indicators conditioned on other urban quantities. In this respect, it is interesting to note the similarities between Colombia and Brazil.

**Figure 6:**
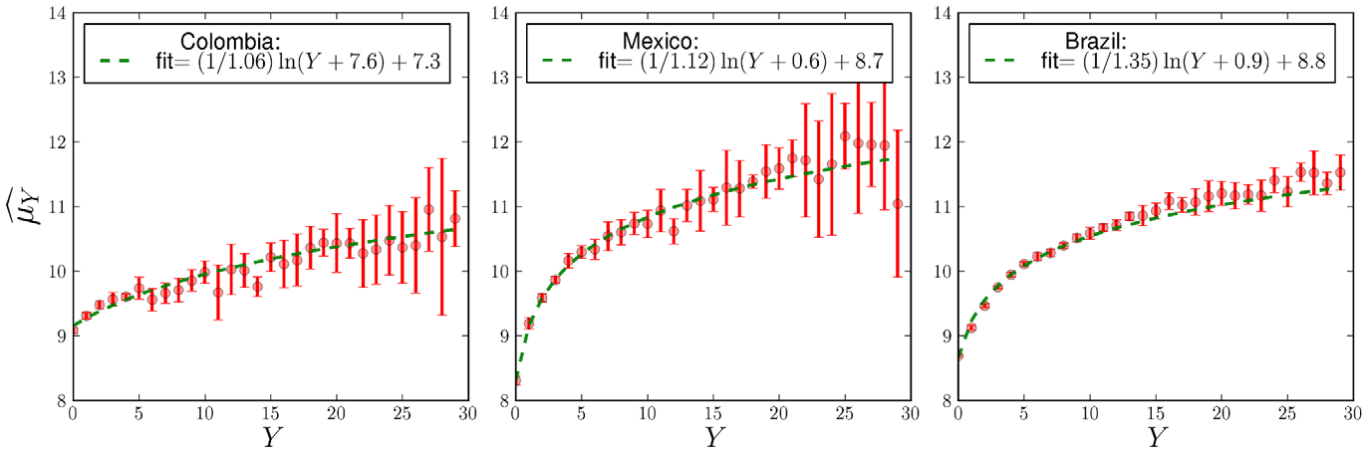
Estimates of 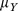 (via maximum likelihood) for different values of 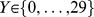, for Colombia, Mexico, and Brazil. A different curve was constructed for every year of the analysis, and the points plotted are the averages over several years. The error bars represent one standard deviation intervals about the mean. Plots show a logarithmic dependence on 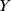, from which a scaling relationship emerges in terms of expectation values (see text). Best fits were obtained using a Levenberg-Marquardt algorithm, weighting every point by its error, see Methods.

We can now estimate the parameters of Eq.(5) using Eqs.(8) and (9), and plot 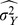 (see Fig. 5) and 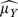 (see Fig. 6) versus 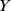, to infer their functional 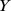-dependence.

The behavior of 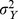 shown in Fig. 5 is stable and we will assume it to be constant henceforth. Because of this we can reject other count models such as the Negative Binomial, which is designed to model over-dispersed data. The curves shown in Fig. 6 display a logarithmic growth of 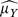 on 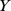. The most general logarithmic function that can be fit to 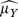 (see Fig. 6) is.
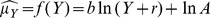
(10)where 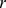 is a positive constant that allows the logarithm to remain finite (and positive) as 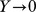 and 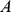 is a positive number. Below, the constant 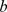 will be identified with the scaling exponent 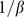. This is the reason why the values of these parameters in Figs. 1 and 6 coincide. The rest of the paper rests on these two assumptions about the behavior of 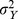 and 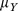, suggested by Figs. 5 and 6. For the fitting procedure of the remaining parameters see the Methods section.

Finally, using Eq.(2), we derive the conditional probability function 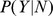. If Eq.(5) holds for all 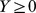, using Eq.(3), we obtain.
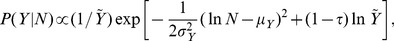
(11)where 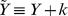.

Using Eq.(10) to replace 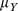 for 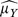, we obtain
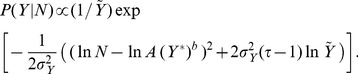
(12)


We can expand the squared terms, the logarithms, group some of the terms, so this equation transforms into:
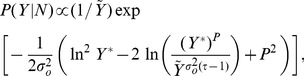
(13)where 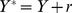, 
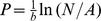
 and 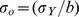.

Now, recall that both 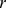 in 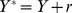 and 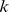 in 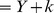 were introduced to account for the limit when 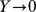. These constants generate the expected limits and prevent us from dividing by zero in the power-law distribution and from taking the logarithm of zero in 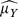. There are no constraints that keep us from assuming them to be equal (and from considering them to be small). Indeed, both introduce a characteristic scale which manifests itself as a regime change in the scaling behavior when cities are very small and realizations of zero homicides (or other discrete measures) begin to occur. Therefore, it is not unreasonable to assume they are they same, thus 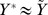 (see the Methods section for an estimation of 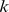). Under this assumption we can complete the square and compute the posterior distribution. Realizing that 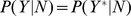 because 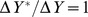, and keeping only 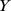 dependent terms (the others will ultimately be absorbed by the normalization constant), we arrive at
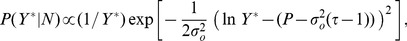
(14)which is a log-normal distribution for 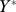 given 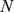, with parameters 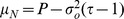 and 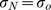. By expressing the distribution parameters in the original variables, and by introducing the proper normalization constant, we finally obtain


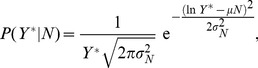
(15)


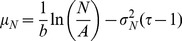
(16)


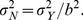
(17)

### The Connection Between Log-normal Statistics, Urban Scaling and Zipf’s Law

These expressions connect the log-normal statistics of the conditional distribution 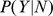 with scaling and Zipf’s law for the size distribution of cities. As we show below this leads to a relationship between scaling and Zipf’s exponents.

Determining these conditional distributions enables us to calculate their moments, such as the mean and variance. We take Eq.(10) and Eq.(16) to derive 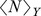 and 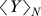 explicitly in terms of 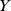 and 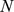, that is:
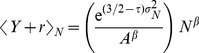
(18)

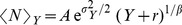
(19)where 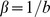 is recovered as the exponent of the scaling relation in Eq.(1). These two expressions represent complementary scaling relations. Note that they are not identical statistically as they express the expectation value of each variable in terms of a given value of the other, not its mean.

Similarly, the standard deviations 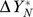 and 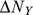 can be expressed as
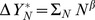
(20)

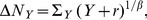
(21)where 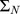 and 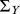 are proportionality coefficients.

From the preceding sections it should already begin to be clear how the log-normal distribution relates to Zipf’s law. We can show how a power-law distribution emerges by deriving the probability distribution of 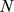. In Eq.(2), 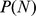 is called the “evidence”, and acts in practice as a normalization constant. It can be calculated from knowledge of the numerator as.
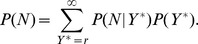


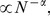
(22)which is a power-law distribution. It then follows that the various exponents are constrained to obey the relationship (see Methods section)



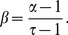
(23)Thus, from this perspective Zipf’s law follows from 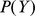 and the statistics of 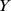 for cities of a given size and the observation of scaling of its expectation value.

If superlinear scaling (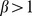) holds for some urban indicator 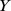, we can predict population sizes to be power-law distributed with exponent 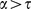, and vice versa if the scaling is sublinear. If 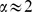 (unity in a rank-size plot) as has been observed for several urban systems [21], superlinear scaling means that 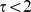, and thus the quantity 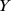 may lack a definite mean and variance. In these cases, references to “average cities” have no sound mathematical meaning. Note however that it is also possible that 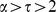 provided that Zipf’s exponent is sufficiently larger than 2, as has been argued long ago by Mandelbrot [22] in a different context. In general these properties can be used to constrain the value of Zipf’s exponent from the observation of the statistics of many different urban indicators and knowledge of their average scaling properties.

## Discussion

In this manuscript we characterized the statistics of homicides - a highly variable and granular metric - in three fast changing urban systems in Latin America. This analysis allowed us to address the statistics of urban indicators under extreme conditions and investigate how urban scaling laws emerge for noisy and granular variables within a larger probabilistic context.

We have found that homicides 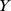 occurring in cities of Brazil, Colombia and Mexico all follow statistics that are well described by log-normal distributions. These distributions are parameterized by an expectation value that is population size dependent and a variance of the log-variables that is not (or that at least can be assumed not to be, for the data analyzed here). In this context scaling laws emerge as the expectation value of 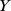 as a function of 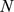, 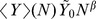. This average relationship, when expressed in terms of logarithms, exposes the issue that it cannot hold in the limit of 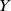 or 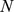 going to zero (unless they do so together). We have devoted particular attention to this regime and found that effectively annual homicide rates saturate at a very small but non-zero value at sufficiently small 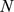. In this sense true scale invariance emerges only when 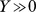. A dual scaling law for 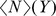 emerges from a Bayesian inversion of the relationship for 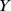 and we have shown that this - i.e. the estimation of 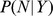 at small discrete 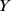 - is often the most practical way to estimate 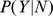. This lead us in turn to the consideration and estimation of 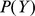 - the distribution of the total number of homicides across cities - which we found to have a Zipfian form. Because these distributions can be used to derive Zipf’s law through marginalization, we obtained a relationship between urban indicator statistics, urban scaling laws and Zipf’s distribution in the form of a constraint between the scaling exponent and the Zipfian exponents for 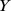 and 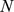.

Much effort has traditionally been devoted to model the broad distributions and lack of characteristic scales displayed by urban systems [23]–[25]. However, our results show that parts of the urban system manifest greater predictability than is usually recognized. Although non-broad distributions would be expected to arise for many quantities when considering cities of fixed size, log-normal statistics are special because they point to multiplicative processes. If these processes depend on the structure of social interactions, log-normality then suggests that quantities should scale with city size in non-trivial ways.

Furthermore, the consistency between log-normal statistics for individual cities and Zipfian distributions for the urban system, as well as scaling relations across city sizes, suggest that local indicators are the result of self-consistent urban system dynamics and that these indicators are naturally bounded. Consequently, when considering goals for urban planning it is important to think at once locally and at the level of the urban system. In this light, on the one hand, questions about particular cities and the magnitude of their metrics may not make much sense unless we take into account the whole urban system in which they are embedded. On the other hand, characterizing urban systems only through power-law distributions prevents us from observing finer quantitative patterns present locally. Several mechanisms have been proposed for the emergence of log-normal [26] and power-law [19], [26]–[28] statistics, usually relying on multiplicative random processes [29], [30]. Population size dependent stochastic interaction processes within cities, which are multiplicative, provide a natural setting to explain these observations and will be the focus of future research.

Given the general implications of these results a few remaining issues and some caveats are worth further discussion. First of all, we motivated the log-normal distribution as a good general description of the data. However, the data may be compatible with other statistical densities, specifically a Laplace distribution (which is also characterized by two parameters a scaling mean and a fixed dispersion, see [14]). We found no consistent evidence in our empirical analysis that pointed conclusively to the need for these alternative and potentially more complex statistical models, but such need may arise as larger datasets are analyzed.

Second, one of our main results is the observation of deviations from scaling in the limit 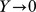, where we are also dealing with small municipalities in terms of 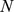. This regime and its statistical treatment is fraught with empirical difficulties, including the fact that we are then dealing predominantly with rural territories in which several small towns are aggregated together as a municipality. Thus, these units are not true single cities. To address this point more disaggregated data would be necessary to probe the behavior of 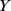 in truly small towns. In this sense our parametrization of the several distributions through the introduction of a saturating constant should be seen as provisional, and is in any case not unique. Another issue, that becomes important for small cities, is the use of annual homicides. If in reality the expected homicide rate vanishes only with vanishing population size, but becomes very small in small towns then it will take on average a longer and longer period of time for any homicides to be observed and any chosen time period will lead to an underestimation of such a rate for a suitably small city. Thus, by making the time period that defines the homicide rate longer we should see the saturating parameters decrease and scale invariance be restored to smaller and smaller population scales. We probed this regime empirically and indeed observed a systematic reduction in the size of cities with zero homicides, but the full consideration of this question is complex and is beyond the current analysis. The empirical and theoretical consideration and re-analysis of these issues may become possible in the future and would be interesting to pursue in order to investigate the limits of urban scaling in small population agglomerations. While it seems plausible to us that a finite probability of violence exists in human communities of any size, the lower limit may be difficult to probe in practice.

As they stand the present results suggest several interesting new questions for future research. First, they provide a mesoscopic view of urban indicators and take a step in suggesting the form of a statistical mechanics approach to universal aggregate properties of cities, such as scaling laws and size distributions. Such an approach should lead to theory and methods to bridge scales of analysis from individuals, through social and economic organizations, to entire cities and urban systems.

**Figure 7:**
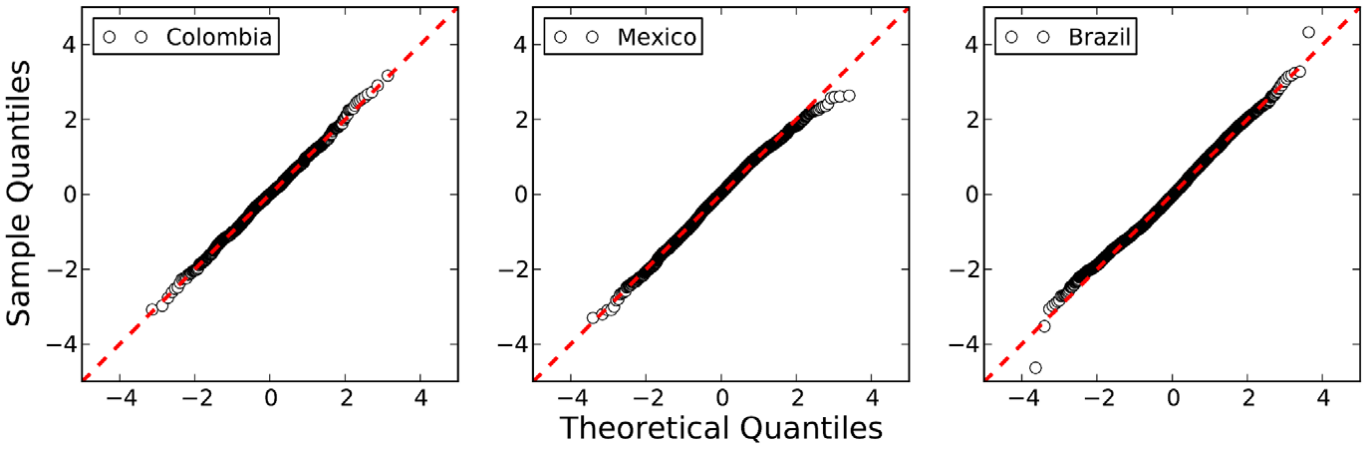
Q-Q plot of the standardized log-variables of the populations of the cities for several values of *Y*. This shows that a log-normal distribution is an excellent description of 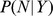, for the three nations, notwithstanding a number of small exceptions at the extremes (a perfect straight line in the dots would correspond to an exact normal distribution of log-populations).

Finally, it is interesting to briefly discuss the practical implications of the statistical treatment of urban indicators developed here. Quantitative knowledge of the distribution of indicators for a given population size allows us to make predictions e.g. for the homicide rate of a particular place with quantified levels of uncertainty. The approach developed here takes into account only data aggregated over a time period, usually a year. However we know in addition that there is also considerable predictability for urban indicators of the same city across time. Thus, we expect that the future combination of these two elements will yield a procedure to make better predictions of future indicators for specific places with quantified uncertainty. This ability will also allow the detection of exceptional events as statistical anomalies in urban indicators. We hope therefore that our growing quantitative understanding of cities and urban systems throughout the world will provide the basis for the development of a predictive science of cities that will help inform more effective policy in an increasingly urbanized world.

## Materials and Methods

### Data Sources

Homicides are defined as deaths caused by other persons, intentionally or not. Data for Colombia is available online at the National Institute of Legal Medicine and Forensic Sciences (http://www.medicinalegal.gov.co) and municipality populations at the National Administrative Department of Statistics (http://www.dane.gov.co). Brazil’s population and homicide numbers are available from the Sangari Institute and Brazilian Ministry of Justice (http://www.sangari.com/mapadaviolencia/). The data spans the years 2003–2007 for Brazil, 2004–2009 for Colombia, and 2005–2009 for Mexico. Data for Mexican municipalities was compiled by Diego Valle [9].

**Figure 8:**
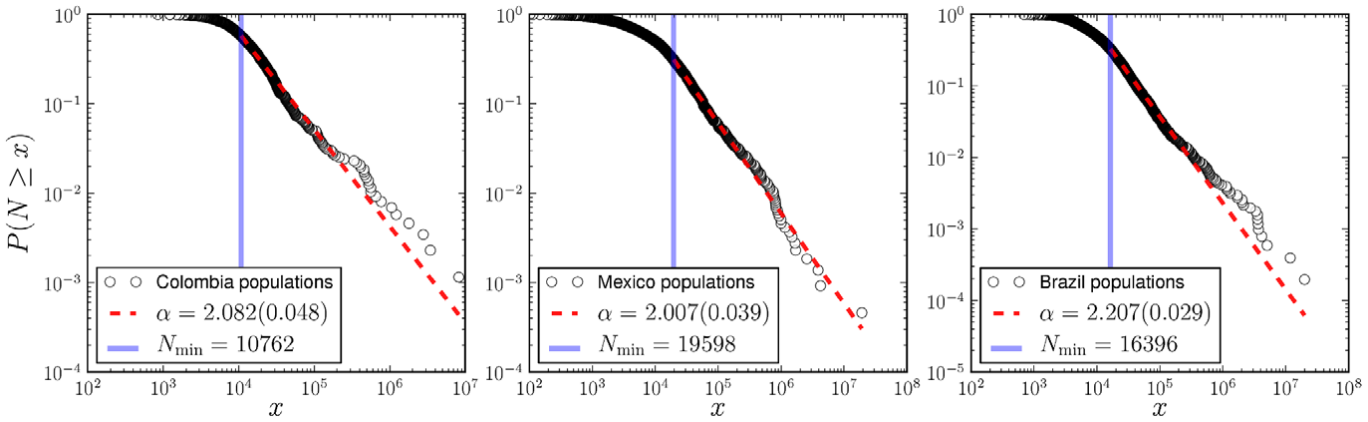
Cumulative normalized distributions of city populations in Colombia, Mexico and Brazil (2007) fitted with pure-power-law distributions. Best fits (dashed red line) of the form 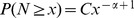 were estimated using the procedure in [17] to the density function. Not disregarding the long-held debate about the city-size distribution, we believe the fit to a power-law distribution stands as a first approximation consistent with our proposed statistical framework.

We adopted standard definitions of metropolitan areas available at http://www.secretariasenado.gov.co/senado/basedoc/ley/1994/ley_0128_1994.html for Colombia. However, comprehensive definitions for many metropolitan areas in Colombia do not exist officially although they are recognized in various contexts (see for example http://www.dane.gov.co/files/censo2005/resultados_am_municipios.pdf for the case of Bogotá). We adopted such unofficial definitions in our analysis. For Mexico definitions are available at the National Institute of Statistics and Geography (http://www.inegi.gob.mx/est/contenidos/espanol/metodologias/otras/zonas_met.pdf), and for Brazil at the Observatory of the Metropolis (http://www.observatoriodasmetropoles.ufrj.br/metrodata/ibrm/index.html).

### Power-law Fits

Reference [17] developed a methodology to estimate the parameters of a power-law fit, and to calculate its associated goodness of fit. The function fitted is the pure power-law
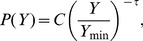
(24)where 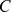 is the normalization constant. The distributions of homicides analyzed here were fitted using this functional form and we were unable to reject the power-law fit. However, the fit only holds for values of 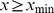. Following [17], the estimated 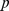-values were 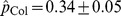, 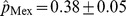 and 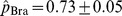, which were not sufficiently small for the power-law distribution to be rejected.

Because we are interested in the regime of small numbers where the number of homicides 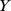 can be zero, we extend Eq.(24) to.
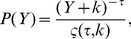
(25)which converges to Eq.(24) for large 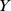, but with the difference that now 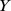 can take any non-negative value.

If we let 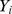, 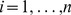, be the observed annual number of homicides of each city. Assuming independence, the log-likelihood of the data under Eq.(25) is
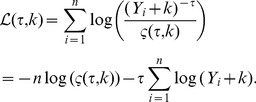
(26)


A numerical estimation of 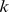 and 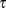 by setting 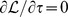 and 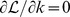 to maximize the likelihood function, yields.
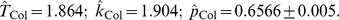


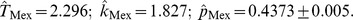


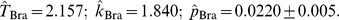



A rigorous procedure to estimate these parameters from the data, estimate the error and determine its scaling properties is part of future work.

### Log-normal Fits

We test the log-normal distribution as a description of 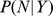 by standardizing the variables 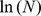 for each given 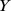, and then showing a normal probability plot (or Q-Q plot) in Fig. 7. Departures from the log-normal distribution (a normal in logarithmic variables) can be identified by departures from the straight line and are shown, in Fig. 7, to be both rare and small.

### Parameter Estimation of 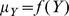


Maximum likelihood estimations of 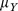 for the different values of 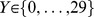, for Colombia, Mexico, and Brazil, are shown in Fig. 6. We constructed a different curve 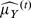 for every year of the analysis, and plotted its average 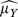 over the set of annual estimates:
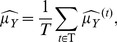
(27)where 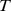 the number of years for which we have data, for each nation.

Error bars represent plus and minus one standard deviation about the average.
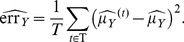
(28)


The fits were performed using a Levenberg-Marquardt algorithm, which minimizes the sum of least squares of a set of non-linear equations, weighting every point by its error. The function to minimize with respect to vector parameter 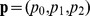 is.
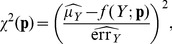
(29)where


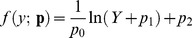
(30)


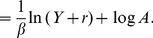
(31)

### Zipf’s Law Derivation

Here we give additional details of the calculations leading to Eq.(22). First, we write 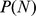 in terms of 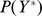 and 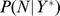:
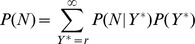


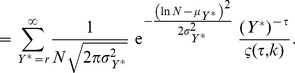
(32)


For simplicity of notation, we drop the subscript in 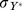, and we use the letter 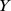, although it is important to keep in mind that we are implicitly referring to 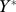. Replacing the sum with an integral and assuming 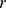 is sufficiently small that we can integrate over the whole range of non-negative numbers, we obtain
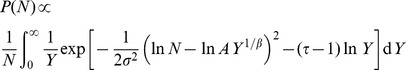


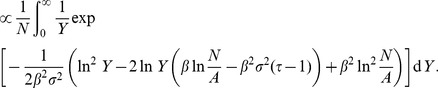
(33)


We can now complete the square and re-arrange terms to obtain.
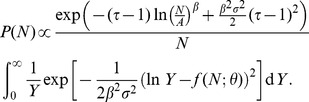
(34)


Now, we see that the term inside the integral is a log-normal distribution, integrated over its entire domain. Consequently, the integral is a constant, regardless of the form of 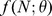, where 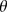 represent the parameters 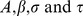. Retaining only terms in 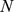, we obtain
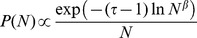


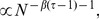
(35)from which we finally see that



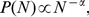
(36)with 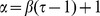, or
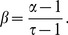
(37)


This relationship can also be derived in a more straightforward way under the assumptions that i) a power-law distribution for 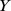 (or 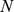) holds and ii) the scaling relationship 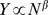 holds *exactly*. Then, using the fact that 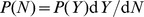, we obtain the same relation between exponents. The derivation given above, however, does not assume an exact expression in the form of 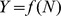, but rather a probabilistic relation between 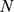 and 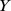, through the expectation value 
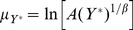
.

Figure 8 shows the cumulative empirical distributions of city populations.
